# Role of the ubiquitin/proteasome system on ACTH turnover in rat corticotropes

**DOI:** 10.1007/s12020-018-1573-9

**Published:** 2018-03-13

**Authors:** Antonella Sesta, Maria Francesca Cassarino, Francesco Cavagnini, Francesca Pecori Giraldi

**Affiliations:** 1 Neuroendocrinology Research Laboratory, Istituto Auxologico Italiano IRCCS, Cusano Milanino (Milan), Milan, Italy; 2 Department of Clinical Sciences and Community Health, University of Milan, Milan, Italy

**Keywords:** Proopiomelanocortin, ACTH, Ubiquitin-proteasome, Ubiquitylation

## Abstract

**Purpose:**

A large number of studies has investigated proopiomelanocortin processing in anterior pituitary corticotropes but little is known on proopiomelanocortin/ACTH degradation within these cells. The ubiquitin-proteasome system is an intracellular protein degradation pathway which has garnered considerable interest in recent times, given its role in maintenance of protein homeostasis. Aim of the present study was to evaluate the role of the ubiquitin-proteasome system in proopiomelanocortin/ACTH turnover in pituitary corticotropes.

**Methods:**

Rat anterior pituitary primary cultures were treated with 0.01–100 nM MG132, a proteasome inhibitor, or 0.1–100 nM K48R, an inhibitor of polyubiquitylation, for 4 and 24 h and ACTH concentrations in medium and cell lysates estimated by immunometric assay. Co-immunoprecipitation for ubiquitin and ACTH was carried out to establish ubiquitin-tagged protein products.

**Results:**

Inhibition of proteasome-mediated degradation with MG132 lead to an increase in ACTH concentrations, both as regards secretion and cell content. Likewise, inhibition of polyubiquitylation was associated with increased ACTH secretion and cell content. Ubiquitin/ACTH co-immunoprecipitation revealed that proopiomelanocortin was a target of ubiquitylation.

**Conclusions:**

We provide the first evidence that the ubiquitin-proteasome system is involved in proopiomelanocortin/ACTH degradation in corticotropes. Indeed, proopiomelanocortin is a target of ubiquitylation and modulation of ubiquitin-proteasome system affects ACTH turnover. This study shows that regulation of ACTH proteolytic degradation may represent a means to control ACTH secretion.

## Introduction

ACTH is a major component of the hypothalamo-pituitary adrenal axis and, thus, pivotal to survival. ACTH is synthesized in anterior pituitary corticotropes upon processing of its precursor, proopiomelanocortin (POMC) and indeed *POMC* null mice [1] or patients carrying a mutation in the *POMC* gene [2] have severe hypocortisolism. POMC, a 241-aminoacid prohormone, is synthesized in the rough endoplasmic reticulum, sorted in the Golgi complex and processed to 39-aminoacid ACTH in secretory granules by prohormone convertase 1/3 (PC1) and cathepsin L [3–5]. ACTH then awaits in mature granules of the regulated secretory pathway until secretion is triggered by specific stimuli [6].

On the other hand, intracellular proteolysis also contributes to active peptide concentrations [7, 8] and eukaryotic cells possess two main proteolytic systems, the vacuolar-lysosomal and the ubiquitin-proteasome system (UPS). The latter, in particular, is deputized to removal of damaged or misfolded proteins, i.e., protein quality control, degradation of short half-life peptides [9, 10] and regulation of intracellular levels of de novo synthesized proteins [11, 12]. Interest in the UPS proteolytic system increased considerably in recent years as impairment in UPS function has been implicated a variety of degenerative diseases, including Parkinson and Alzheimer, as well as neoplasias, e.g., breast cancer [7, 13–16]. Degradation of proteins by the ubiquitin-proteasome system is accomplished in two steps: mono/polyubiquitylation of the target protein followed by proteolytic degradation of the ubiquitylated protein by the 26 S proteasome macromolecular complex [9]. Ubiquitin is attached to its substrate through an enzymatic cascade, comprising an ubiquitin-activating enzyme (E1), an ubiquitin conjugase (E2) and an ubiquitin ligase (E3). These enzymes conjugate the substrate onto ubiquitin via its lysine residues, i.e., ubiquitylation, and, given that ubiquitin contains 7 lysine residues, consecutive rounds of ubiquitylation can result in the formation of long and diverse ubiquitin chains [9, 17]. The tagged protein is then anchored to the 26 S proteasome and degraded and free, reusable ubiquitin released.

Aim of the present study was to evaluate the role of ubiquitin-proteasome system on ACTH turnover in pituitary corticotropes. Our study identified POMC as a target of ubiquitylation and showed that inhibitors of ubiquitylation and of the ubiquitin-proteasome system increased ACTH cell content, as well as secretion. It follows, therefore, that ubiquitylation is directly involved in regulation of intracellular ACTH homeostasis.

## Materials and methods

### Rat anterior pituitary primary cultures

Anterior pituitaries were obtained by dissection from adult male Sprague-Dawley rats (*rattus norvegicus*, Charles River Laboratories, Calco, Italy) maintained in light-dark cycle and temperature-controlled rooms with free access to laboratory chow and tap water. Animals were treated according to the National Institutes of Health, Office of Animal Care and Use recommendations and authorization from the University of Milan Animal Care offices was obtained prior to the study. Pituitaries were established in culture using our usual protocol [18, 19]. Briefly, anterior pituitaries were excised, dispersed with trypsin, cell dispersions pooled and cells plated at 4-5 × 10,000 cell/well density in 12-well polystyrene plates (Corning Inc., Corning NY, USA). Primary cultures were attached in Dulbecco’s modified Eagle’s medium (DMEM), containing glucose and L-glutamine supplemented with 10% fetal bovine serum and antibiotics and maintained at 5% CO_2_, 37 °C for 3-5 days.

### Treatments

Rat anterior pituitary primary cultures were incubated in serum-free DMEM + 0.1% bovine serum albumin (BSA) containing 0.01–100 nM carbobenzoxy-L-leucyl-L-leucyl-L-leucinal MG132, a peptide aldehyde which selectively inhibits chymotrypsin-like proteolysis and the ubiquitin-proteasome pathway [12, 20], or 0.1–100 nM mutant ubiquitin K48R, an inhibitor of polyubiquitylation [21]. Co-treatments with 5 µM cycloheximide, an inhibitor of protein synthesis and translational elongation, and 0.01–10 nM MG132 were also performed. Control wells were treated with DMEM and 0.1% BSA alone and each treatment was performed in quadruplicate. After 4 and 24 h, medium was collected and cell content extracted. Parallel assessments for cell viability were performed with trypan blue staining [22]. All reagents were obtained from Sigma-Aldrich, St. Louis MO, USA and stock solutions dissolved according to the manufacturer’s instructions, i.e., MG312 in DMSO, K48R in sterile water. Experiments were repeated at least thrice.

### Co-immunoprecipitation and Western blotting

Total protein was extracted from control wells by RIPA Lysis buffer (25 mM Tris-HCl pH 7.6, 150 mM NaCl, 1%NP-40, 1% sodium deoxycholate, 0.1% SDS; Thermo Scientific, Rockford IL, USA) supplemented with protease and phosphatase inhibitor cocktail (Sigma Aldrich, St. Louis MO, USA). Protein concentration was measured using Bradford assay (BioRad, Hercules CA, USA). For co-immunoprecipitation experiments, cell lysates (150 µg) were incubated at 4 °C overnight with anti-ubiquitin rabbit polyclonal primary antibody (1:2000 dilution; Abcam, Cambridge, UK) and non immune IgG (i.e., non specific control). Protein complexes were captured on Protein A/G PLUS-agarose (Santa Cruz Biotechnology Inc. Dallas TX, USA). Ubiquitin-precipitated pituitary primary culture cell extracts were separated on SDS-PAGE using a 4-12% gradient (NuPage gel in Tris-glicine, Life Technologies, Carlsbad CA, USA) under denaturing conditions. Proteins bands were transferred to Hybond ECL nitrocellulose membrane (GE Healthcare, Little Chalfont, UK) and the membrane blocked with 5% non-fat milk, incubated with anti-ACTH rabbit polyclonal primary antibody raised against the entire 1-39 sequence (1:1000 dilution; Abcam, Cambridge, UK) followed by incubation with horseradish peroxidase-conjugated secondary goat polyclonal anti-rabbit antibody (1:10000 dilution; Invitrogen, Camarillo CA, USA). Blots were developed using enhanced chemiluminescence technique. Bioinformatic prediction of ubiquitylation sites on rat POMC protein sequence (GenBank Accession # AAH58443) was perfomed at www.ubpred.org.

### Assays

ACTH in cell extracts and media was measured by immunoradiometric assay (Diasorin S.p.A. Saluggia, Italy). This sandwich assay uses two antibodies, one specific to ACTH 1-17 and the other to ACTH 26-39, thus measures intact ACTH 1-39. No interference with POMC is expected given assay methodology. Intraassay coefficient of variation is 7.9%, and assay sensitivity 1.2 pg/ml. Responses were normalized to percent of control secretion (unstimulated secretion = 100%). Prolactin in cell extracts and media was measured by immunoradiometric assay (Institute of Isotopes, Budapest, Hungary). Intraassay coefficient of variation is 3.2%, and assay sensitivity 0.07 ng/tube.

### Statistical analysis

Statistical comparisons were performed using ANOVA followed by Fisher’s PLSD post-hoc test. Statistical significance accepted at *p* < 0.05. Data are described as mean ± S.E.M.

## Results

**Fig. 1:**
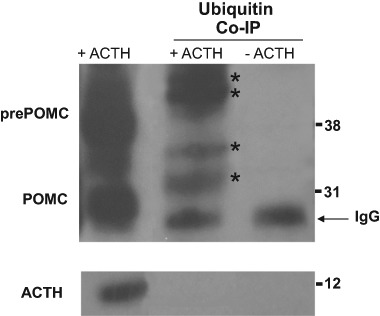
Ubiquitin/ACTH co-immunoprecipitation in rat anterior pituitary primary cell extracts. Left lane shows input blotted for ACTH: prePOMC (∼39 kDa), POMC (∼29 kDa) and ACTH (∼6 kDa) are visible. Middle lane shows ubiquitin-tagged ACTH-blotted fragments: Asterisk (*) identfies two bands for ubiquitylated POMC (i.e., ∼32 kDa non-glycosylated and ∼37 kDa glycosylated POMC) and prePOMC ubiquitylated fragments (∼45 kDa and over). No band was observed at the expected size for ubiquitylated ACTH. Right lane shows ubiquitin immunoprecipitation without ACTH blotting: arrow identifies IgG light chains (~ 23 KDa) visible in both middle and right lanes

First, we sought to determine targets of ubiquitylation by ubiquitin/ACTH co-immunoprecipitation. Western blotting on ubiquitin-precipitated cell extracts showed that only the POMC precursor was ubiquitylated as no band corresponding to ACTH was detected in ubiquitin-tagged precipitates (Fig. 1). As expected, ubiquitylated POMC and prePOMC presented higher molecular weight compared to native POMC by approx. 8 kDa corresponding to ubiquitin moieties. Both isoforms of POMC, i.e., non-glycosylated and glycosylated fragments, appeared to be targets of ubiquitylation as was the 267-aminoacid prePOMC precursor. The search for canonical ubiquitylation sites on the rat POMC sequence revealed 4 potential lysine residues at 76, 122, 163, and 184.

**Fig. 2:**
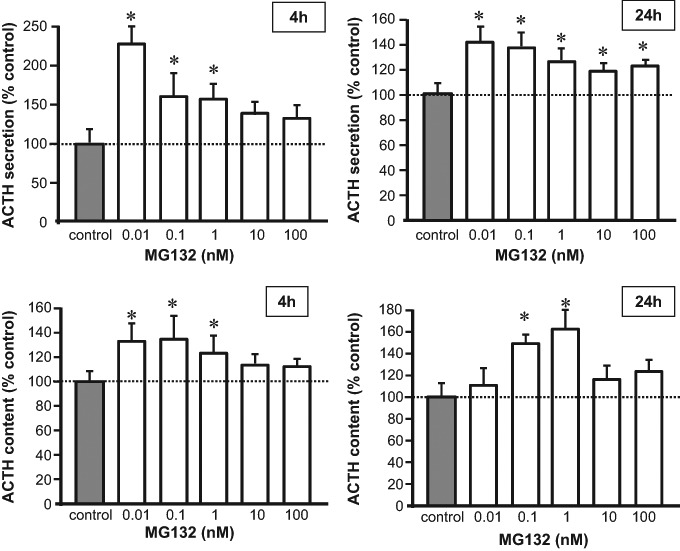
ACTH concentrations in medium and cell content in rat anterior pituitary primary cell cultures treated with 0.01–100 nM MG132 for 4 h and 24 h (white bars). Each treatment was carried out at least thrice on quadruplicate 4-5 × 10,000 cells/well. Dashed line represent unchallenged wells set at 100% (control; gray bar). Asterisk (*) denotes *p* < 0.05 vs. control as assessed by Fisher’s PLSD post-hoc test

We then evaluated the effects of MG132, an inhibitor of degradation by proteasome, on ACTH levels and observed an increase in ACTH concentrations in medium and cell content. The increase in ACTH secretion was evident both 4 h and 24 h (ANOVA *F* = 2.446, *p* < 0.05 and *F* = 3.857, *p* < 0.01, respectively), up to twice as high with 0.01 nM MG132 at 4 h (Fig. 2). The increase in cell content was significant at both timepoints (F = 5.031, *p* < 0.005 and *F* = 3.261, *p* < 0.05, for 4 h and 24, respectively) up to 50% of unchallenged wells (Fig. 2). Specificity of MG132 proteasome inhibition on POMC/ACTH was assessed by measuring prolactin, as prolactin secretion is known not to be affected by proteasome inhibitors.[23] Indeed, prolactin levels in cell extracts and medium did not change following MG132 treatment (medium: 110.3 ± 6.81% control; extract: 94.5 ± 9.69% control after 24 h incubation, N.S.). No changes in cell viability were observed during MG132 incubation (Supplementary Table 1) thus observed effects are not due to toxicity.

**Fig. 3:**
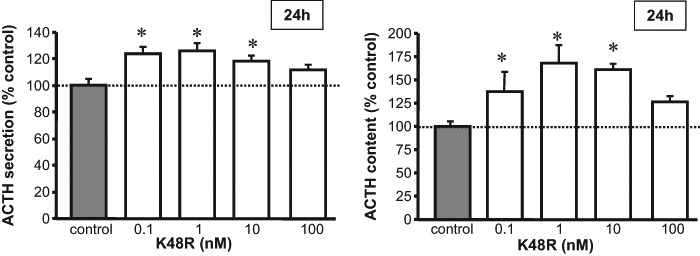
ACTH concentrations in medium and cell content in rat anterior pituitary primary cell cultures treated with 0.1–100 nM K48R for 4 h and 24 h (white bars). Each treatment was carried out at least thrice on quadruplicate 4–5 × 10,000 cells/well. Dashed line represent unchallenged wells set at 100% (control; grey bar). Asterisk (*) denotes *p* < 0.05 vs. control as assessed by Fisher’s PLSD post-hoc test

Incubation with K48R, an inhibitor of polyubiquitylation, also led to a significant increase in ACTH medium concentrations after 24 h (*F* = 5.504, *p* < 0.05; Fig. 3). Likewise, ACTH cell content was increased during 24 h incubation with K48R (*F* = 3.550, *p* < 0.05; Fig. 3). No significant effect was observed after 4 h K48R incubation in either medium (*F* = 0.401, N.S.) or cell content (*F* = 1.498, N.S.) Lastly, no changes in cell viability were observed during incubation with K48R, (Supplementary Table 1) again attesting to lack of toxicity at the doses tested.

**Fig. 4:**
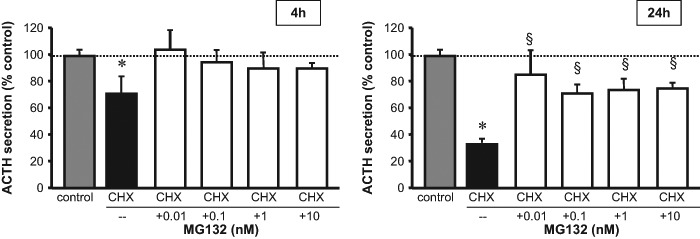
ACTH concentrations in medium in rat anterior pituitary primary cell cultures treated with 5 µm cycloheximide (CHX; black bar) and 0.01–10 nM MG132 for 4 and 24 h (white bars). Each treatment was carried out at least thrice on quadruplicate 4–5 × 10,000 cells/well. Dashed line represent unchallenged wells set at 100% (control; grey bar). Asterisk (*) denotes *p* < 0.05 vs. control; ^§^*p* < 0.05 vs. cycloheximide as assessed by Fisher’s PLSD post-hoc test

In order to establish whether the proteasome acts upon newly synthetized ACTH, we performed co-incubation experiments with 5 µM cycloheximide. ACTH medium concentrations were decreased by some 70% after 24 h incubation with cycloheximide (*F* = 278.18, *p* < 0.001; Fig. 4); as in the previously-described experiments without cycloheximide, MG132 co-incubation brought about an increase in ACTH concentrations (*F* = 8.827, *p* < 0.01, Fig. 4) indicating that proteasome inhibition acts in absence of de novo synthetized ACTH. Inhibition by cyclohexamide was less evident at 4 h (*F* = 6.907, *p* < 0.05) and the counteracting effects of MG132 did not reach significance (*F* = 0.979, N.S.; Fig. 4). No effect of cycloheximide incubation on cell content was observed at either timepoint (4 h: *F* = 1.293, N.S.; 24 h: *F* = 0.210, N.S.) thus no counteracting effect of MG132 could be observed (4 h: *F* = 0.318, N.S.; 24 h: *F* = 0.455, N.S.).

## Discussion

Our study provides the first demonstration that the POMC peptide is degraded by the ubiquitin-proteasome system (UPS) in corticotrope cells and that modulation of the UPS system directly affects ACTH turnover and secretion. Indeed, while there is a considerable body of studies on POMC processing to ACTH and other POMC-derived peptides, such as ß-lipotropin, α- and ß-melanocyte-stimulating hormones, little is known on degradation of POMC or its derived peptides.

The ubiquitin-proteasome system is a highly selective cytosolic protein degradation mechanism [9] whose main function is removal of abnormal proteins, which may prove toxic if accumulated, and of rapidly acting regulatory proteins, whose short half-lives have evolved to facilitate regulation of their activity [10, 17, 24]. Thus, it participates in wide array of cellular processes including cell signaling, cell cycle progression, differentiation and apoptosis [9]. Derangement of UPS has been implicated in neurodegenerative disorders such as Parkinson’s disease, [13] Alzheimer’s disease [25], in nephrotic disease [26] and, importantly, neoplasia [15]. In fact, proteasome inhibitors are a relatively new target-treatment class for cancer [12] and some compounds, e.g., bortezomib, have already been approved for use in hematologic malignancies and offer promise for solid tumors [14, 16].

In physiological conditions, the ubiquitin-proteasome system together with the lysosomal apparatus subserve protein degradation to maintain appropriate intracellular protein stores, the so-called “proteostasis” [17]. The importance of proteases in maintaining overall intracellular ACTH metabolism has previously been shown for cysteine and aspartyl proteases [27]. In our study, we demonstrate for the first time that the UPS is involved in corticotrope POMC/ACTH turnover.

We observed a clear increase in ACTH concentrations in intracellular stores and incubation medium during incubation with MG132, an UPS-protease inhibitor. Similar effects were observed with the mutant ubiquitin K48R, an inhibitor of polyubiquitylation, which proves that attachment of ubiquitin chains is required for POMC/ACTH degradation. In order to identify targets of ubiquitylation, we performed ubiquitin/ACTH co-immunoprecipitation studies and can report that proteins in the molecular weight range of POMC and prePOMC were ubiquitylated whereas ACTH itself did not appear a target of ubiquitylation. Of note, the POMC peptide contains four lysine residues -canonical ubiquitin target sites- and an abundance of cysteines, serines and threonines which may represent alternative ubiquitylation sites [28].

Given that POMC is transported from the endoplasmic reticulum to the transGolgi network [3, 6], it is likely that POMC is subject to endoplasmic reticulum-associated degradation (ERAD), a process that allows proteins to be back-transported out of the endoplasmic reticulum to the cytoplasm and thus degraded by cytoplasmic UPS [29, 30]. ERAD was initially discovered as a mechanism for removal of misfolded proteins from the endoplasmic reticulum but was subsequently also demonstrated to occur also for regulated proteins [11, 31]. POMC would therefore be targeted by ubiquitin ligases located in endoplasmic reticulum membrane and delivered to the cytoplasm via the retrotranslocation complex [31]. Any change in ACTH, which is formed downstream to the endoplasmic reticulum, would thus be secondary to ubiquitylation of its prohormone prior to PC1 cleavage [3, 6]. On the other hand, ubiquitylation has recently been demonstrated to promote also lysosomal sorting [32], thus ubiquitylated POMC could also be directed towards lysosomal degradation. In fact, protein substrate sorting to either proteasomes or lysosomes depends on ubiquitylated lysines, as well as length and type of polyubiquitin chain branching [33]. Given that MG132 and K48R clearly increased ACTH concentrations, ubiquitin-mediated lysosomal degradation may come into play as an additional proteolytic process.

An increase in ACTH secretion was observed already after 4 h incubation with MG132, suggesting reduced degradation of the ready releasable pool of POMC/ACTH [6]. Indeed, the effect of MG132 was evident also during cyclohexamide blockade, indicating that UPS-proteases do not require newly synthesized ACTH, but act upon protein moieties already present within the cell. In this context, although co-immunoprecipitation experiments proved that POMC is a direct target for ubiquitylation, additional factors may come into play given the complexity of POMC synthesis and processing [3, 6]. Inhibition of ubiquitylation may interfere with proteolytic degradation of any factor involved in POMC-to-ACTH processing, e.g., PC1, cathepsin L [4, 6], thus leading to increased activity of these enzymes. Of note, MG132 has been shown to inhibit proteolysis of mutated PC1 [34] but whether the convertase is target of ubiquitin-mediated proteasomal degradation in physiological conditions remains to be seen. In this context, it is worth noting that the effect of both agents on ACTH homeostasis was more pronounced in the pico- and low nanomolar range and less in the high nanomolar range. This suggests that MG132 and K48R act upon several intracellular ubiquitylation targets -possibly with contrasting functions- which ultimately affect ACTH synthesis/secretion.

Our findings on the role of UPS in POMC/ACTH turnover are of particular interest given the recent reports on gain-of-function mutations in the thiol protease deubiquitinase *USP8* gene in patients with ACTH-secreting pituitary adenomas, i.e., Cushing’s disease [35–37]. Deubiquitinases are enzymes which remove ubiquitin moieties from a given substrate thus steering proteins tagged for proteolysis away from their intended fate [17]. Mutations in the *USP8* 14-3-3 binding motif lead to increased catalytic activity [35, 36] and *USP8* mutants result in increased deubiquitination of ligand-activated epidermal growth factor (EGF) receptor [35, 36], a factor involved in tumoral corticotrope pathophysiology [38, 39]. Ultimately, *USP8* mutants lead to inhibition of EGF signaling downregulation and increased *Pomc* expression and ACTH secretion [35, 36]. In addition to this effect of tumoral corticotrope secretory activity, UPS also appear involved in tumoral corticotrope proliferation as silencing of cullin4A, a core subunit of E3 ubiquitin ligase, led to decreased proliferation of AtT-20 cells [40].

In conclusion, our study provides evidence that the POMC peptide is degraded by the ubiquitin-proteasome pathway and that inhibition of ubiquitylation increases ACTH concentrations. These results show that modulation of the UPS affects ACTH turnover in corticotrope cells and pave the way to novel avenues of research in both normal and neoplastic ACTH-secreting cells.

## Electronic supplementary material

Supplementary Table 1(DOC 29 kb)
