# Supplementary material for: Role of the ubiquitin/proteasome system on ACTH turnover in rat corticotropes
Source: Endocrine. 2018 Mar 13;61(3):511–7. doi: 10.1007/s12020-018-1573-9 (PMC6105253; doi:10.1007/s12020-018-1573-9)
Supplement: Supplementary file 1 — Supplementary Table 1(DOC 29 kb) [file 12020_2018_1573_MOESM1_ESM.doc]

Supplementary Table 1. Cell viability assessed by Trypan blue staining

| **MG132** |  |
| --- | --- |
| 0.01 nM | 99.3 ± 5.47 % control |
| 0.1 nM | 95.2 ± 4.66 % control |
| 1 nM | 98.6 ± 8.23 % control |
| 10 nM | 103.4 ± 7.22 % control |
| 100 nM | 92.5 ± 6.42 % control |
| **K48R** |  |
| 0.1 nM | 110.9 ± 16.67 % control |
| 1 nM | 108.7 ± 15.94 % control |
| 10 nM | 95.8 ± 3.62 % control |
| 100 nM | 105.2 ± 9.42 % control |
